# The Shark Basal Hypothalamus: Molecular Prosomeric Subdivisions and Evolutionary Trends

**DOI:** 10.3389/fnana.2018.00017

**Published:** 2018-03-14

**Authors:** Gabriel N. Santos-Durán, Susana Ferreiro-Galve, Arnaud Menuet, Sylvie Mazan, Isabel Rodríguez-Moldes, Eva Candal

**Affiliations:** ^1^Grupo BRAINSHARK, Departamento de Bioloxía Funcional, Universidade de Santiago de Compostela, Santiago de Compostela, Spain; ^2^UMR7355, CNRS, University of Orleans, Orleans, France; ^3^CNRS, Sorbonne Université, Biologie Intégrative des Organismes Marins, UMR7232, Banyuls-sur-Mer, France

**Keywords:** chondrichthyan, basal hypothalamus, evo-devo, prosomeric model, segments, Shh, PCNA

## Abstract

The hypothalamus is a key integrative center of the vertebrate brain. To better understand its ancestral morphological organization and evolution, we previously analyzed the segmental organization of alar subdivisions in the catshark *Scyliorhinus canicula*, a cartilaginous fish and thus a basal representative of gnathostomes (jawed vertebrates). With the same aim, we deepen here in the segmental organization of the catshark basal hypothalamus by revisiting previous data on *ScOtp, ScDlx2/5, ScNkx2.1, ScShh* expression and Shh immunoreactivity jointly with new data on *ScLhx5, ScEmx2, ScLmx1b, ScPitx2, ScPitx3a, ScFoxa1, ScFoxa2 and ScNeurog2* expression and proliferating cell nuclear antigen (PCNA) immunoreactivity. Our study reveals a complex genoarchitecture for chondrichthyan basal hypothalamus on which a total of 21 microdomains were identified. Six belong to the basal acroterminal region, the rostral-most point of the basal neural tube; seven are described in the tuberal region (Tu/RTu); four in the perimamillar region (PM/PRM) and four in the mamillar one (MM/RM). Interestingly, the same set of genes does not necessarily describe the same microdomains in mice, which in part contributes to explain how forebrain diversity is achieved. This study stresses the importance of analyzing data from basal vertebrates to better understand forebrain diversity and hypothalamic evolution.

## Introduction

The hypothalamus is an important physiologic center of the brain. It integrates information from limbic, endocrine and autonomic sources to elaborate different kinds of homeostatic and behavioral responses such as feeding or reproduction. Its organization has been elusive for neuroanatomists as result of complex patterning processes converging at this point (Shimamura et al., 1995; Puelles and Rubenstein, 2003; Puelles et al., 2004; Medina, 2008; Szabó et al., 2009; Shimogori et al., 2010; Alvarez-Bolado et al., 2012; Beccari et al., 2013; Croizier et al., 2015).

The prosomeric model, a modern paradigm of vertebrate brain development and organization (Puelles and Rubenstein, 2003, 2015; Puelles et al., 2012; Puelles, 2017), understands the hypothalamus to be located ventral to the telencephalon, being both located rostral to the diencephalon (or primary prosencephalon). Moreover, telencephalon and hypothalamus (known together as secondary prosencephalon) are subdivided into two true segments: hp2, rostral or terminal; hp1, caudal or peduncular (see Puelles et al., 2012). The intrahypothalamic border (IHB) separates hp2 from hp1 while the hypothalamo-diencephalic border (HDB) separates hp1 from p3, the rostral-most unit of the tripartite segmental diencephalon (Puelles et al., 2012).

The updated prosomeric view of the hypothalamus understands it to be organized into five longitudinal histogenetic domains dorso-ventrally arranged into two alar and three basal domains (Puelles et al., 2012; Puelles and Rubenstein, 2015) separated by the alar-basal boundary (ABB). Moreover, these dorso-ventral domains can be further subdivided into two rostro-caudal subdomains (terminal or peduncular; the last also indicated by the particle “retro”): terminal and peduncular paraventricular area (TPa/PPa); terminal and peduncular subparaventricular area (TSPa/PSPa); tuberal and retrotuberal area (Tu/RTu); perimamillary and periretromamillary area (PM/PRM); mamillary and retromamillary area (MM/RM; Morales-Delgado et al., 2011, 2014; Puelles et al., 2012; Díaz et al., 2015; Ferrán et al., 2015; Rodríguez-Moldes et al., 2017). Furthermore, at the rostral-most hp2, where the alar and basal plates meet, a region referred as acroterminal is recognized. It has special patterning properties that are at the basis of the development of structures like the optic chiasm or the neurohypophysis (Puelles et al., 2012; Ferrán et al., 2015; Puelles and Rubenstein, 2015). Noteworthy, the underlying logic of segments, boundaries, histogenetic domains, subdomains and microdomains proposed by the prosomeric framework rely on conserved molecular mechanisms (Puelles and Rubenstein, 1993, 2003, 2015; Puelles and Medina, 2002; Puelles et al., 2012; Puelles, 2017). As a result, the prosomeric framework became key for homologies establishment and is largely accepted as a comparative tool (Puelles and Rubenstein, 2003; Martínez-de-la-Torre et al., 2011; Medina et al., 2011; Moreno et al., 2012, 2017; Domínguez et al., 2013, 2014, 2015; González et al., 2017; Pombal and Megías, 2017; Rodríguez-Moldes et al., 2017).

Cartilaginous fishes, also known as Chondrichthyans, are a key group for evo-devo studies. They are among the most basal extant groups of gnathostomes (jawed vertebrates) being the closest out-group to osteichthyans (the other major phylum of gnathostomes, which includes bony fishes and tetrapods). Therefore, they are essential to address the ancestral condition of the vertebrate brain (Coolen et al., 2009). In previous work, we sketched prosomeric organization in the catshark *Scyliorhinus canicula* to better understand the ancestral condition of the vertebrate hypothalamus (Santos-Durán et al., 2015). In this study, the expression of *ScNkx2.1, ScDlx2/5, ScShh* and *ScOtp* led to the identification of alar and basal domains, apparently homologous to the murine ones. However, this work also suggested that alar organization seems to be more conserved than basal one, what correlates with the development of conserved and divergent adult structures, respectively (Santos-Durán et al., 2015; Rodríguez-Moldes et al., 2017). In subsequent work we deeply tested prosomeric assumptions in the alar hypothalamus on the light of additional makers. Our findings suggested conserved traits that can be traced back to the agnathan-gnathostome transition (Santos-Durán et al., 2016). Now, we revisit the organization of the basal hypothalamus with similar aims: (i) to look for further prosomeric molecular subdivisions; (ii) to test if new data on gene expression patterns support previous prosomeric interpretations; and (iii) to obtain some insights on the evolution of this region by comparative analysis. Noteworthy, conserved adult structures (i.e., tracts of the hypothalamic-hypophyseal system, a median eminence or the neurohypophysis) and divergent ones (i.e., inferior hypothalamic lobes and the *saccus vasculosus*) emerge from this territory offering an attractive scenario for evolutionary insights. To address these questions, previous data on *ScNkx2.1*, *ScDlx2/5*, *ScOtp, ScShh* expression and Shh immunoreactivity were revised jointly with new data on *ScLhx5*, *ScEmx2*, *ScLmx1b*, *ScPitx2, ScPitx3a, ScFoxa1, ScFoxa2 and ScNeurog2* expression and proliferating cell nuclear antigen (PCNA) immunoreactivity patterns. Here we were able to identify a plethora of subdomains (microzones) in the catshark hypothalamus. A comparative analysis of microzone identity in catshark is made with mammals but not with other vertebrates due to the lack of detailed data. However gross comparisons among vertebrates prompt the idea that the caudal border of the hypothalamus, as it is currently defined, could be a derived character rather than a conserved one, a feature that deserves further investigation.

## Materials and Methods

### Experimental Animals

Some embryos of the catshark (lesser spotted dogfish; *S. canicula*) were supplied by the Marine Biological Model Supply Service of the CNRS UPMC Roscoff Biological Station (France). Additional embryos were kindly provided by the Aquaria of Gijón (Asturias, Spain), O Grove (Pontevedra, Spain) and Finisterrae (A Coruña, Spain). Embryos were staged by their external features according to Ballard et al. (1993). For more information about the relationship of embryonic stages with body size, gestation and birth, see Table 1 in Ferreiro-Galve et al. (2010). Sixty-nine embryos from stages 28 to 32 were used in this study. Eggs from different broods were raised in seawater tanks in standard conditions of temperature (15–16°C), pH (7.5–8.5) and salinity (35 g/L). Adequate measures were taken to minimize animal pain or discomfort. All procedures conformed to the guidelines established by the European Communities Council Directive of 22 September 2010 (2010/63/UE) and by the Spanish Royal Decree 53/2013 for animal experimentation and were approved by the Ethics Committee of the University of Santiago de Compostela.

### Tissue Processing

Embryos were deeply anesthetized with 0.5% tricaine methane sulfonate (MS-222; Sigma, St. Louis, MO, USA) in seawater and separated from the yolk before fixation in 4% paraformaldehyde (PFA) in elasmobranch’s phosphate buffer [EPB: 0.1 M phosphate buffer (PB) containing 1.75% urea, pH 7.4] for 48–72 h depending on the stage of development. Subsequently, they were rinsed in phosphate buffer saline (PBS), cryoprotected with 30% sucrose in PB, embedded in OCT compound (Tissue Tek, Torrance, CA, USA), and frozen with liquid nitrogen-cooled isopentane. Parallel series of sections (12–20 μm thick) were obtained in transverse planes on a cryostat and mounted on Superfrost Plus (Menzel-Glasser, Madison, WI, USA) slides.

### Single and Double Immunohistochemistry on Sections and Whole Mounts

For heat-induced epitope retrieval, sections were pre-treated with 0.01 M citrate buffer (pH 6.0) for 30 min at 95°C and allowed to cool for 20–30 min at room temperature (RT). Sections were then rinsed twice in 0.05 M Tris-buffered saline (TBS; pH 7.4) for 5 min each and incubated overnight with the primary antibody (polyclonal rabbit anti-Sonic Hedgehog [anti-Shh], Santa Cruz Biotechnology, Santa Cruz, CA, USA, diluted 1:300; monoclonal mouse anti-proliferating cell nuclear antigen [anti-PCNA] Sigma, St. Louis, MO, USA, diluted 1:500). Appropriate secondary antibodies (horseradish peroxidase [HRP]-conjugated goat anti-rabbit and anti-mouse, BIORAD, diluted 1:200) were incubated for 2 h at RT. The immunoreaction was developed with 0.005% diaminobenzidine (DAB; Sigma) and 0.003% H_2_O_2_ for 20–40 min. Sections were rinsed in distilled water (twice for 30 min), allowed to dry for 2 h at 37°C and mounted in MOWIOL 4-88 Reagent (Calbiochem, MerkKGaA, Darmstadt, Germany). All dilutions were made with TBS containing 15% donkey normal serum (DNS; Millipore, Billerica, MA, USA), 0.2% Triton X-100 (Sigma) and 2% bovine serum albumin (BSA, Sigma).

Whole mounts embryos were prepared for IHC as previously described in Santos-Durán et al. (2015). After fixation with 4% PFA in 0.01 M PBS at 4°C for 2 days, embryos were washed in 0.9% NaCl in distilled water, dehydrated in graded series of methanol solutions (50%, 80%, 100%) and stored at −20°C. Samples to be stained were placed on ice in 2 ml of dimethyl sulfoxide (DMSO)/methanol (1/1) until they sank. Then, 0.5 ml of 10% Triton X-100/distilled water was added, and the embryos were incubated for 30 min at RT. After washing in 0.05 M TBS with 0.1% Triton X-100 (TST, pH 7.4), samples were sequentially blocked using spin-clarified aqueous 1% periodic acid and 5% non-fat dried milk in TST (TSTM). Primary antibody (polyclonal rabbit anti-Sonic Hedgehog [anti-Shh], Santa Cruz Biotechnology, Santa Cruz, CA, USA, diluted 1:300) was diluted in TSTM containing 0.1% sodium azide for 2–4 days at RT with gently agitation on a shaking platform. The secondary antibody (horseradish peroxidase [HRP]-conjugated goat anti-rabbit, BIORAD, diluted 1:200 in TSTM) was incubated overnight. After a final washing in TST, the embryos were pre-incubated with 0.25 mg/mL diaminobenzidinetetrahydrochloride (DAB, Sigma) in TST with 2.5 mg/mL nickel ammonium sulfate for 1 h, and then allowed to react with DAB in TST containing 2.5 mg/mL nickel ammonium sulfate and 0.00075% H_2_O_2_ for 20–40 min at RT. The reaction was stopped using Tris-HCl buffered saline and specimens were post-fixed with 4% PFA overnight at 4°C. Epidermis and mesodermic derivatives were carefully removed and specimens were rinsed in graded series of glycerol (25%, 50%, 75% and 100%) and observed under the stereomicroscope.

### Controls and Specificity of the Antibodies

No immunostaining was detected when primary or secondary antibodies were omitted during incubations. The monoclonal anti-PCNA antibody specifically labels proliferating cells in the brain, retina and olfactory epithelium of this species (Rodríguez-Moldes et al., 2008; Ferrando et al., 2010; Ferreiro-Galve et al., 2010; Quintana-Urzainqui et al., 2014). The polyclonal anti-Shh antibody (Santa Cruz Biotechnology Inc., Santa Cruz, CA, USA) was raised in rabbit against the amino acids 41–200 of the human Shh protein. We previously reported that the *in situ* hybridization (ISH) results were similar to those obtained by IHC, and therefore validate the specificity of the anti-Shh antibody used here (Santos-Durán et al., 2015).

### *In Situ* Hybridization on Sections and Whole Mounts

We applied *in situ* hybridization for *ScOtp* (Quintana-Urzainqui, 2013; Santos-Durán et al., 2015, 2016), *ScDlx2* (Quintana-Urzainqui et al., 2012, 2015; Compagnucci et al., 2013; Debiais-Thibaud et al., 2013; Quintana-Urzainqui, 2013; Santos-Durán et al., 2015, 2016)*, ScDlx5* (Compagnucci et al., 2013; Debiais-Thibaud et al., 2013; Santos-Durán et al., 2015, 2016), *ScNkx2.1* (Quintana-Urzainqui et al., 2012; Quintana-Urzainqui, 2013; Santos-Durán et al., 2015, 2016), *ScLhx5* (Santos-Durán et al., 2016), *ScEmx2* (Derobert et al., 2002), *ScLmx1b* (Pose-Méndez et al., 2016), *ScPitx2* (Lagadec et al., 2015), *ScPitx3a*, *ScFoxa1*, *ScFoxa2* and *ScNeurog2* (Santos-Durán et al., 2016) genes. These probes were selected from a collection of *S. canicula* embryonic cDNA library (mixed stages S9–S22), constructed in pSPORT1, and submitted to high throughput EST sequencing. Selected cDNA fragments were cloned in pSPORT vectors. Sense and antisense digoxigenin-UTP-labeled and fluorescein-UTP-labeled probes were synthesized directly by *in vitro* transcription using as templates linearized recombinant plasmid DNA or cDNA fragments prepared by PCR amplification of the recombinant plasmids. *In situ* hybridization in whole mount and on cryostat sections was carried out following standard protocols (Coolen et al., 2009). Briefly, sections were permeabilized with proteinase K, hybridized with sense or antisense probes overnight at 65°C and incubated with the alkaline phosphatase-coupled anti-digoxigenin and anti-fluorescein antibody (1:2000, Roche Applied Science, Manheim, Germany) overnight at 4°C. The color reaction was performed in the presence of BM-Purple (Roche). Control sense probes did not produce any detectable signal.

### Image Acquisition and Analysis

Light field images were obtained with an Olympus BX51 microscope equipped with an Olympus DP71 color digital camera. Photographs were adjusted for brightness and contrast and plates were prepared using Adobe Photoshop CS4 (Adobe, San Jose, CA, USA).

## Results

### *ScNkx2.1* and *ScDlx2/ScDlx5* Expression. Comparison With *ScShh*-Expression/Shh Immunoreactivity

An overview of the expression of *ScShh*, *ScNkx2.1, ScOtp* and *ScDlx2*/*ScDlx5* in the basal hypothalamus of *S. canicula*, mainly in early stages of development, has been previously described in Santos-Durán et al. (2015). The location of the ABB was re-examined in Santos-Durán et al. (2016).

Here we revisited these data to deepen in the genoarchitectonic profile of the basal hypothalamus and further characterize possible dorso-ventral and rostro-caudal subdomains of this territory. A detailed comparative analysis of the expression of such genes in sagittal and transverse sections is presented from stages 29 to 32, when the basic mature cytoarchitecture and organization of the adult hypothalamus are clearly recognized.

#### *ScShh*-Expression/Shh Immunoreactivity

**Figure 1:**
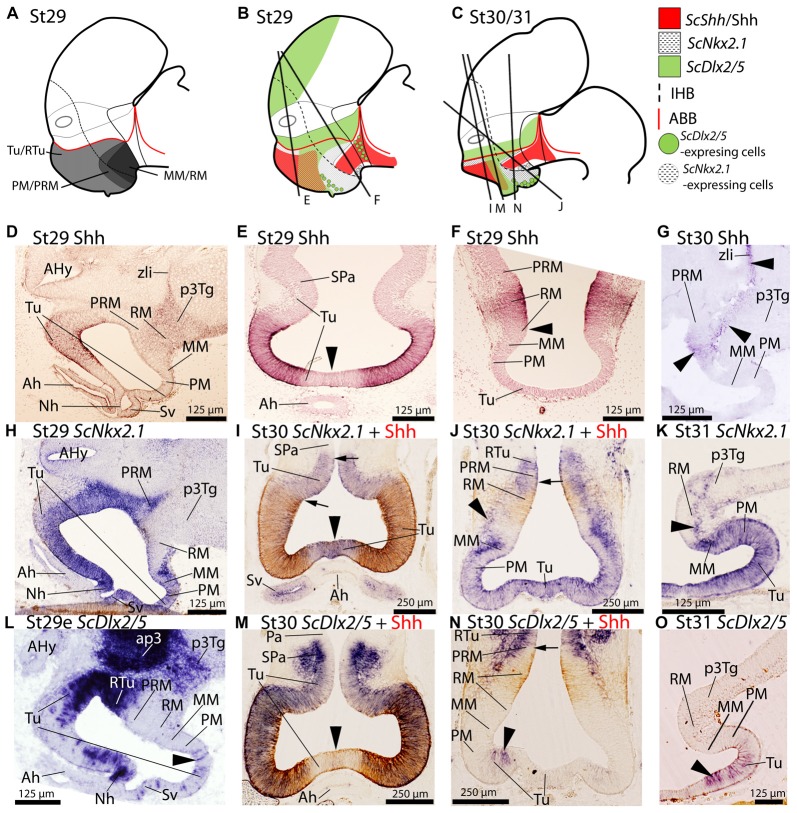
Regionalization of the basal hypothalamus and neighbor territories in embryos of *S. canicula* at stages 29–31. Sagittal schemes **(A–C)** and sections showing immunoreactivity to Shh **(D–G)**, and expression of *ScNkx2.1* **(H–K)**, and *ScDlx2/5* **(L–O)** by means of immunohistochemistry (IHC) **(D–G)**, and *in situ* hybridization **(H–O)** on sagittal **(D,G,H,K,L,O)** or transverse **(E,F,I,J,M,N)** sections. Some *in situ* sections were double labeled for IHC against Shh **(I,J,M,N)**. **(A–C)** Schemes of basal hypothalamus compartments at parasagittal levels at stage 29 and stage 30/31. For simplicity, the schemes do not represent medial (acroterminal) expression patterns. **(A)** Shark basal hypothalamic compartments at stage 29 as defined in Santos-Durán et al. (2015). **(B,C)** Expression patterns of ScShh/Shh, ScNkx2.1 and ScDlx2/5 at stage 29 and 30/31. **(D–G)** Shh immunoreactivity in the Tu and RM. Note also the continuity of labeling along the p3Tg and the zona limitans intrathalamica (zli). Black arrowhead in **(E)** points the lack of labeling in the acroterminal region. Arrowhead in **(F)** marks the absence of Shh immunoreactivity in the ventral-most portion of RM. Arrowheads in **(G)** show weak Shh immunoreactivity in RM, p3Tg and zli at stage 30. **(H–K)** *ScNkx2.1* expression is observed in the different territories of the basal hypothalamus excepting the RM. Black arrowhead in **(I)** points the acroterminal region showing *ScNkx2.1* expression and absence of Shh immunoreactivity. Arrows in **(I)** point differences between the pattern of distribution of Shh immunoreactivity and *ScNkx2.1* expression. Arrowheads in **(J,K)** point *ScNkx2.1*-expressing cells in the RM mantle. Arrow in **(J)** points dorsal-most distribution of Shh immunoreactivity. **(L–O)*** ScDlx2/5* expression in restricted regions of the Tu/RTu. Note also intense labeling in ap3. Arrowheads in **(L,N,O)** indicate dispersed *ScDlx2/5*-expressing cells in the caudo-ventral part of Tu. Arrowhead in **(M)** marks the acroterminal region lacking *ScDlx2/5* expression and Shh immunoreactivity. For abbreviations, see list.

Since *ScShh* detection by means of ISH at early developmental stages yields similar results to those obtained by IHC against anti-Shh (Santos-Durán et al., 2015), here we have used the antibody to analyze additional developmental stages and to ease pattern comparisons by means of double ISH-IHC staining. As described in Santos-Durán et al. (2015); from stage 29 onwards, Shh immunoreactivity is observed in part of the rostral and dorsal Tu domain and broadly detected within the RM domain, extending from here along the diencephalic basal plate (see Figures 1A–F; see also Santos-Durán et al., 2015). Shh immunoreactivity is not observed in the SPa domain (Figures 1B,E; see also Santos-Durán et al., 2016) or in the midline (acroterminal territory) just dorsal to the developing adenohypophysis (black arrowhead in Figure 1E). Caudally, Shh immunoreactivity is only observed in a portion of the RM domain but not at its dorsal-most and ventral-most portions (Figure 1B and arrowhead in Figure 1F). At stage 30, Shh immunoreactivity is still detected in the hypothalamus and primary prosencephalon (arrowheads in Figure 1G; see also Santos-Durán et al., 2015) but it becomes reduced in the RM and basal plate of p3 (p3Tg) compared to previous stages.

#### *ScNkx2.1* Expression

From stage 29 onwards, *ScNkx2.1* is expressed ventral to the optic stalk through the whole basal hypothalamus except in the RM compartment (see Figures 1A–C,H–K). While *ScNkx2.1* expression and Shh immunoreactivity co-distribute in part of the Tu domain (Figures 1C,I), Shh immunoreactivity does not match the dorsal border of *ScNkx2.1* expression (black arrows in Figure 1I). In contrast to Shh, *ScNkx2.1* is additionally expressed in the acroterminal territory dorsal to the adenohypophysis (arrowhead in Figure 1I). *ScNkx2.1* expression in the MM abuts the RM, but it does not meet Shh immunoreactivity since Shh is absent from the ventral-most portion of RM, which creates a gap between both (Figures 1C,J; see also Santos-Durán et al., 2015). Of note, *ScNkx2.1* expression in the ventricular zone forms a clear-cut border between the positive MM and the negative RM domain that is more evident on sagittal sections (Figure 1K), though *ScNkx2.1*-expressing cells can be detected in the mantle of the RM domain (black arrowhead in Figures 1J,K).

#### *ScDlx2*/*ScDlx5* Expression

In the basal plate *ScDlx2/5* is intensely expressed in a restricted subdomain of the Tu/RTu and the p3Tg domains (Figure 1L). In the hypothalamus, it is expressed in a subdomain spreading from the RTu to the neurohypophysis (Figures 1L–N). Rostrally, *ScDlx2/5* expression in the basal hypothalamus co-distributes with Shh immunoreactivity in a subdomain of the Tu (Figures 1C,M). Note that neither *ScDlx2/5* expression nor Shh immunoreactivity can be observed in the acroterminal territory co-extensive with the adenohypophysis (arrowhead in Figure 1M) but it is expressed in the neurohypophysis (Figure 1L). *ScDlx2/5* expression in the caudal-most RTu almost abuts Shh immunoreactivity in the RM although a gap exists (Figure 1C; arrow in Figure 1N). In the most caudo-ventral part of Tu, individual and dispersed *ScDlx2/5*-expressing cells can be recognized almost reaching the rostral and ventral-most part of the PM (arrowheads in Figures 1L,N) including the primordium of the *saccus vasculosus*. These cells are less intensely labeled but still observable at stage 31 (arrowhead in Figure 1O). At stage 32 the basic pattern described for *ScDlx2/5* is maintained although its expression becomes reduced in intensity (see below).

### *ScLhx*5 and *ScOtp* Expression

#### *ScLhx5* Expression

**Figure 2:**
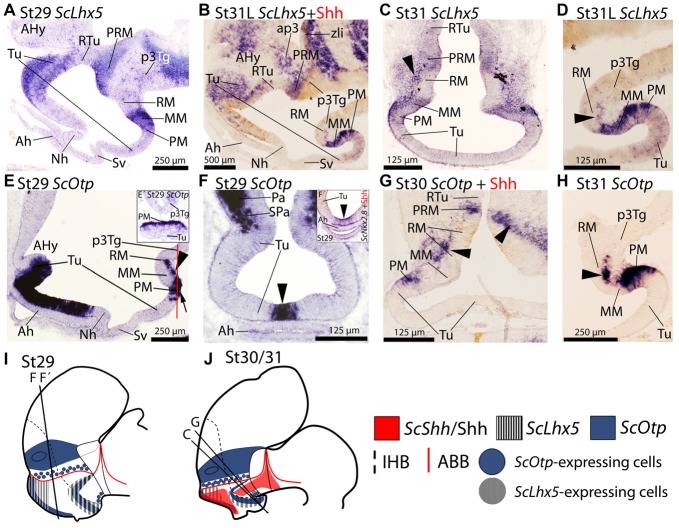
Regionalization of the basal hypothalamus and neighbor territories in embryos of *S. canicula* at stages 29–31. Sections showing expression of *ScLhx5* **(A–D)** *ScOtp* **(E–H)** by means of *in situ* hybridization **(A–H)** on sagittal **(A,B,D,E,H)** or transverse **(C,F,G)** sections. **(I,J)** Sagittal schemes to show expression patterns of *ScShh*/Shh, *ScLhx5* and *ScOtp* at stage 29 **(I)** and stage 30/31 **(J)**. For simplicity, the schemes do not represent medial (acroterminal) expression patterns. Some *in situ* sections were double labeled for IHC against Shh **(B,G)**. **(A–D)** *ScLhx5* expression in the Tu, PM/PRM and MM domains. Arrowhead in **(C)** points *ScLhx5*-expressing cells in the mantle of the RM domain. Arrowhead in **(D)** shows a sharp limit abutting RM. Note that dispersed *ScLhx5* expressing cells in the caudal and ventral-most part of Tu are not represented in **(I,J)**. **(E–H)** *ScOtp* expression in regions of the Tu and PM/PRM domains. Inset **(E′)** shows a transverse section at the level indicated by the red line in **(E)**. Arrowhead in **(F)** points the restricted *ScOtp* expression in the midline of the acroterminal region. Arrowhead in **(F′)** points *ScNkx2.8* expression. Arrowheads in **(E,G,H)** indicate *ScOtp*-expressing cells in the mantle of the RM region lacking Shh immunoreactivity. For abbreviations, see list.

From stage 29 onwards, in the basal plate, *ScLhx5* is observed in a subdomain of the dorsal-most and rostral-most Tu domain (Figures 2A,B,I,J). Dispersed *ScLhx5-*expressing cells are also observed in the most caudo-ventral part of Tu where individual and dispersed *ScDlx2/5*-expressing cells were observed (compare Figure 2D with Figure 1O). *ScLhx5* expression can also be observed in the PM/PRM and MM domains (Figures 2A–D,I,J). Note that *ScLhx5* expression in the MM domain describes a clear-cut border with the RM domain (Figure 2D), though *ScLhx5*-expressing cells can be recognized in the mantle of the RM domain (arrowheads in Figures 2C,D).

#### *ScOtp* Expression

In the basal plate, *ScOtp* has been identified in Tu and PM/PRM domains (Santos-Durán et al., 2015). From stage 29 onwards *ScOtp* is expressed in the rostral-most part of the Tu domain, from the optic stalk to the primordial neurohypophysis (Figure 2E). Specifically, it is restricted to the acroterminal territory of the Tu domain just dorsal to the adenohypophysis (Figures 2E,I; arrowhead in Figure 2F) codistributing with *ScNkx2.8* (arrowhead in Figure 2F′). In the PRM domain, *ScOtp* expression abuts the RM but not the Shh immunoreactivity of this domain (Figures 2G,J). In the PM, *ScOtp* is also expressed in the acroterminal territory (Figure 2E′). Note that the expression of *ScOtp* in the PM faces the MM (Figure 2H). Marginal *ScOtp-expressing* cells can be recognized in the RM (black arrowheads in Figures 2G,H) and p3Tg (not shown). This pattern is maintained until stage 32.

### *ScEmx2* Expression

**Figure 3:**
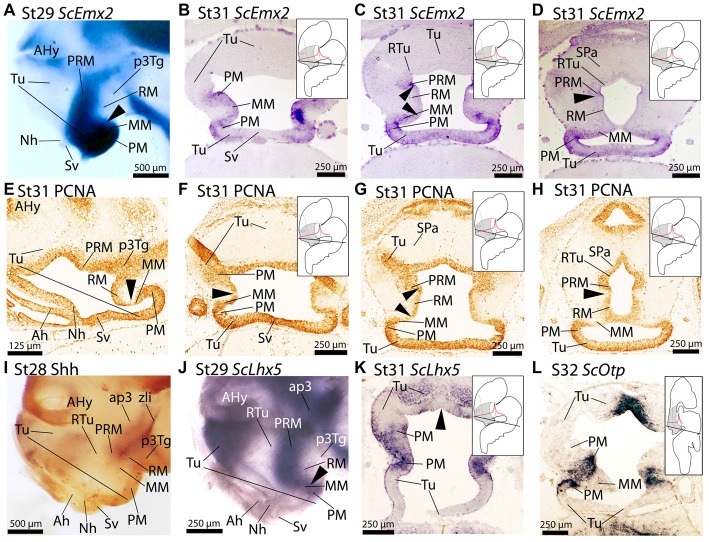
Regionalization of the basal hypothalamus and neighbor territories in embryos of *S. canicula* at stages 29–32 based on the expression of *ScEmx2* **(A–D)**, *ScLhx5* **(J,K)**, *ScOtp* **(L)** and immunoreactivity to proliferating cell nuclear antigen (PCNA) **(E–H)** and Shh **(I)** on whole mounts **(A,I,J)** and in sagittal **(E)** and transverse **(B–D,F–H,K,L)** sections. **(A–D)** *ScEmx2* expression through the caudo-ventral Tu, PM/PRM and MM domains. Note the absence of expression in the RM domain and in the rostral part of the acroterminal territory. **(A)** Lateral view of a whole mount. Arrowhead marks the caudal border of the *ScEmx2* expression in the MM. Note the sharp limit with the negative RM domain. **(B–D)** Sequence of sections from ventral **(B)** to dorsal **(D)** levels of the basal hypothalamus. Arrowheads in **(C)** mark how the *ScEmx2* expression in the MM and PRM borders the negative territory of the RM. Arrowhead in **(D)** points the caudal border of *ScEmx2* expression in the PRM. **(E–H)** PCNA immunoreactivity in sections at equivalent levels to those showed in **(A–D)**. Arrowheads indicate discontinuities in PCNA immunoreactivity. Discontinuities match *ScEmx2* expression borders. **(I)** Lateral view of a whole mount embryo stained for Shh immunoreactivity to show the complementary pattern to that of *ScEmx2*. **(J,K)** Lateral view of a whole mount embryo **(J)** and transverse section **(K)** showing that the caudal domain of *ScLhx5* (corresponding to PM/PRM and MM) codistributes with *ScEmx2* (compare **J** with **A** and **K** with **C**). **(L)** *ScOtp* expression in restricted territories of PM/PRM and MM domains. Compare with expressions of *ScLhx5* in **(K)** and *ScEmx2* in **(C)** to notice that different subdomains can be identified in PM/PRM and MM comparing the expression of these three genes. For abbreviations, see list.

The expression of *ScEmx2* has been analyzed by Derobert et al. (2002) in the brain and related tissues from early stages of development (stage 19) until mid-gestation stages (stages 28–30). Here we analyze in detail the expression of *ScEmx2* in the basal hypothalamus from stage 29 until stage 31. From stage 29 onwards, *ScEmx2* is expressed in the basal hypothalamus in a well-defined domain spreading into part of rostral and ventral-most Tu domain, the PM/PRM and the MM domains (Figures 3A–D). Of note, its expression lacks in midline domains of the Tu (acroterminal territory) such as the neurohypophysis (Figure 3A) and *saccus vasculosus* (Figure 3B) but is present immediately caudal to the last (Figures 3C,D).

A comparison of *ScEmx2* expression with other genes reveals several correlations. We compared *ScEmx2* expression patterns with the presence of PCNA-immunoreactive (-ir) cells (Figures 3E–H). PCNA-ir cells define proliferative zones that are separated by non-proliferative (PCNA-immunonegative) ventricular regions, which are believed to define important segmental boundaries (reviewed in Candal et al., 2005). Of note, the caudal border of *ScEmx2* expression in the MM domain (arrowheads in Figures 3A,E) correlates with a domain of reduced PCNA immunoreactivity in the ventricular zone (arrowheads in Figures 3E–G). The caudal border of *ScEmx2* expression in the PRM domain also correspond with a domain of restricted PCNA immunoreactivity (compare arrowheads in Figures 3D,H). Thus, a band of reduced or negative proliferation seems to spread from the rostral and dorsal border of the RM (Figures 3F–H), p3Tg and zona limitans intrathalamica (zli; not shown).

Finally, we compared *ScEmx2* expression with that of other genes usually expressed in the basal hypothalamus to better understand its organization. A comparison with Shh immunoreactivity (Figure 3I) revealed that the *ScEmx2*-expressing domain in the PRM is fairly complementary to Shh in the RM domain (compare Figures 3A,I). Besides, this *ScEmx2*-expressing domain includes the caudal domain expressing *ScLhx5* in the PM/PRM and MM (compare Figures 3A,J) and *ScOtp* in the PM/PRM (compare Figures 3B,L). Of note, *ScEmx2*, *ScLhx5* and *ScOtp* define consecutively more restricted domains (compare Figures 3B,K,L). Moreover, the expression of *ScEmx2* abuts that of *ScDlx2/5* in dorsal and caudal positions (RTu domain) while they co-distribute in more rostral and ventral positions (not shown).

### *ScLmx1b, ScPitx2, ScPitx3a, ScFoxa1, ScFoxa2* and *ScNeurog2* as Markers of the RM

*ScFoxa1* and *ScFoxa2* are expressed in fairly the same spatial and temporal patters in the regions and stages considered in this study and thus are conjointly referred as *ScFoxa1/2*.

**Figure 4:**
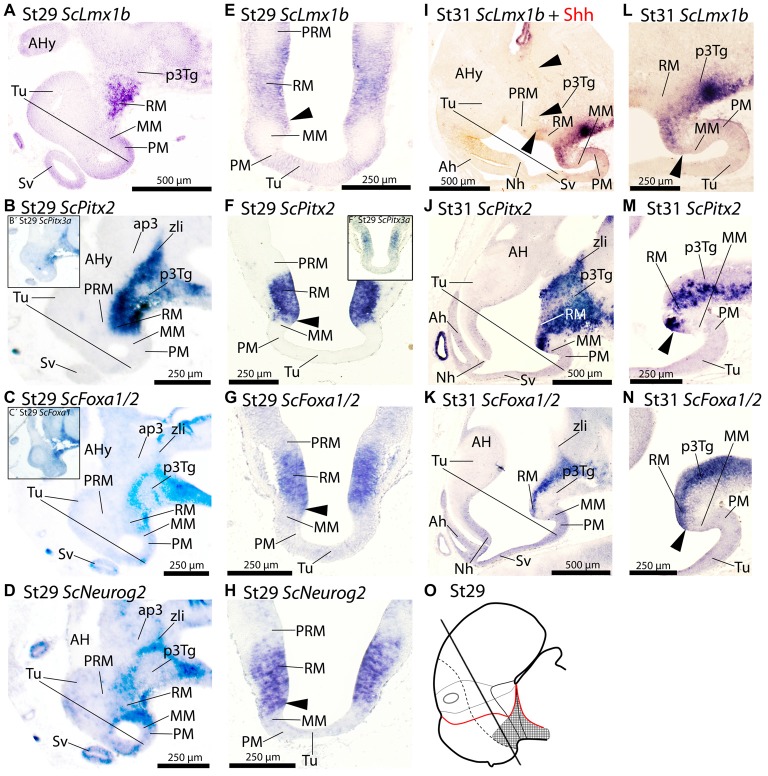
Regionalization of the basal hypothalamus and neighbor territories in embryos of *S. canicula* at stages 29–31 based on the expression of *ScLmx1b* **(A,E,I,L)**, *ScPitx2* **(B,F,J,M)**, *ScPitx3a* **(B′,F′)**, *ScFoxa1/2* **(C,C′,G,K,N)** and *ScNeurog2* **(D,H)** in sagittal **(A–D,I–N)** and transverse **(E–H)** sections. The level of transverse sections is indicated in the scheme **(O)**. For simplicity, the schemes do not represent medial (acroterminal) expression patterns. Some *in situ* sections were double labeled for IHC against Shh **(I)**. **(A–D)** Equivalent sagittal sections of embryos at stage 29 showing the similar pattern of expression of *ScLmx1b, ScPitx2, ScFoxa1*/*2* and *ScNeurog2*. Insets **(B′)** and **(C′)** show similar results when using *ScPitx3a* and *ScFoxa1 probes*. **(E–H)** Transverse equivalent sections showing that, in any case, the expression of *ScLmx1b, ScPitx2a, ScFoxa1*/*2* and *ScNeurog2* is restricted to the RM. Arrowheads points the sharp limit where the expression of these genes is abutting the negative MM. Inset **(F′)** show that *ScPitx3a* expression is similar to that of *ScPitx2* shown in **(F)**. **(I–N)** Panoramic views **(I–K)** and details **(L–N)** of sagittal sections to show similar patterns of expression of *ScLmx1b, ScPitx2a and ScFoxa1*/*2* in the RM. Arrowheads in **(I)** point immunoreactivity to Shh in the RM and zli. Arrowheads in **(L–N)** point the region where the expression of such genes abuts the negative MM. For abbreviations, see list.

At stage 29 *ScLmx1b, ScPitx2, ScPitx3a, ScFoxa1*/*2* and *ScNeurog2* are expressed in a similar pattern spreading caudally from RM into the diencephalon including the zli in the case of *ScPitx2, ScPitx3a, ScFoxa1*/*2* and *ScNeurog2* (Figures 4A–D,B′,C′,O). On transverse sections the expression of these genes dorsally abuts the PRM (Figures 4E–H,F′). Rostrally these genes also abut the MM (arrowheads in Figures 4E–H). From stage 29 onwards this general pattern persists although some differences emerge. At stage 31, *ScLmx1b* becomes downregulated being restricted to the floor plate (Figure 4I) while Shh immunoreactivity is still found in the basal plate (arrowheads in Figure 4I). At stage 31 *ScPitx2* (Figure 4J) is still expressed in the pattern observed at stage 29, while *ScPitx3a* is restrictedly expressed in the caudal-most diencephalon (not shown). In the case of *ScFoxa1/2*, there is slight dorsal and ventral downregulation but the main pattern persists through RM and diencephalon (Figure 4K). From stage 30 onwards, *ScNeurog2* becomes downregulated in the mentioned territories although it can be recognized in the zli and habenulae (data not shown). Finally, *ScLmx1b*, *ScPitx2* and *ScFoxa1/2* still present a sharp border of expression that abuts the MM domain at this developmental stage (arrowheads in Figures 4L–N).

## Discussion

**Figure 5:**
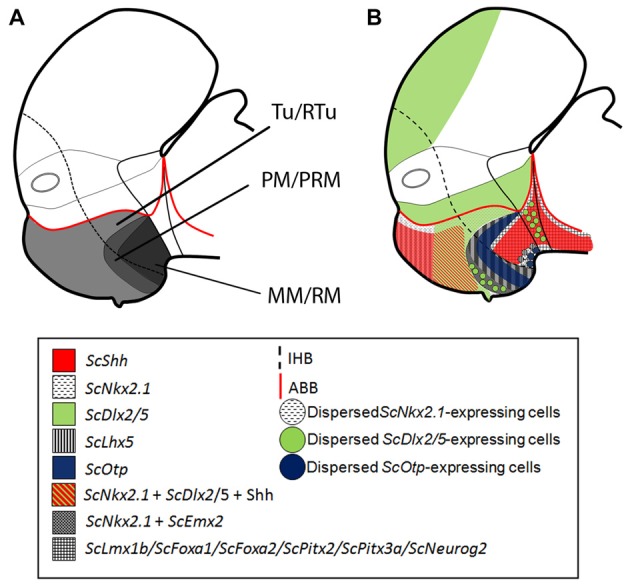
Schematic representation of the catshark basal hypothalamus at mid development. For simplicity, the schemes do not represent medial (acroterminal) expression patterns. **(A)** Representation of prosomeric histogenetic domains Tu/RTu, PM/PRM, MM/RM based on Santos-Durán et al. (2015). **(B)** Genoarchitectonic organization based on current data. Different microzones are recognized into main histogenetic domains. For abbreviations, see list.

**Figure 6:**
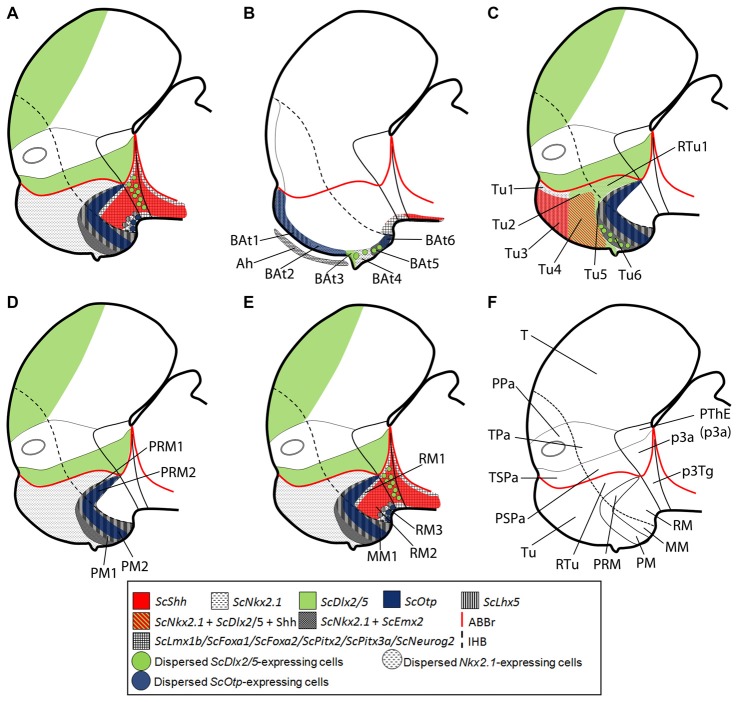
Schematic representation of the catshark basal hypothalamus genoarchitecture. All schemes represent parasagittal sections except **(B)** that represents a medial sagittal section. **(A)** Global organization of catshark hypothalamus. **(B)** Basal acroterminal (BAt) territory and corresponding microzones. The expression of *ScNkx2.1*, *ScOtp*, *ScLhx5*, *ScDlx2/5* and *ScEmx2* defines six microzones (BAt1–6). BAt1–2 are coextensive with adenohypophysis suggesting a signaling influence. BAt3 corresponds to neurohypophysis. BAt4 give rise to *saccus vasculosus*. BAt5–6 are involved in posterior recess organ development. **(C)** Tu/RTu subdomains and microzones. In this territory *ScNkx2.1 is* expressed alone or in combination with *ScLhx5*, *ScDlx2/5, ScEmx2* and Shh immunoreactivity. Seven microzones are identified (Tu1–6 and RTu1). Dispersed *ScLhx5*-expressing cells in Tu6 are not represented. **(D)** PM/PRM subdomains and neighbor territories where *ScNkx2.1* co-distributes with *ScEmx2*. The expression of *ScNkx2.1, ScEmx2, ScLhx5* and *ScOtp* define four subdomains (PM1–2, PRM1–2). **(E)** MM/RM subdomains and microzones. The mamillar subdomain where *ScNkx2.1, ScLhx5* and* ScEmx2* are expressed is represented by MM1. These markers and those expressed in PRM2 define a clear-cut border with *ScLmx1b*, *ScPtix2*, *ScPtix3a*, *ScFoxa1/2* and *ScNeurog2* expression and Shh immunoreactivity in the RM. Their expression is dynamic but three microdomains can be sketched within the RM (RM1–3). Note that almost all genes expressed in the RM are continuously detected into the basal plate of the diencephalon and also in the zli forming a clear-cut border with those rostrally expressed. **(F)** Representation of main territories in the hypothalamus and rostral diencephalon. For abbreviations, see list.

In a previous work, we identified the shark basal hypothalamus harboring three domains (Tu/RTu, PRM/PM and MM/RM) based on the basal expression of *ScNkx2.1*, *ScShh*, *ScOtp* and *ScDlx2/5* (Santos-Durán et al., 2015). In the present work we revisit such analysis on the light of *ScLhx5, ScEmx2, ScLmx1b, ScPitx2, ScPitx3a, ScFoxa1, ScFoxa2* and* ScNeurog2* expression, besides Shh and PCNA immunoreactivity. Different subdomains were identified within the aforementioned domains and within the basal acroterminal region, the basal rostral-most neural tube (Figures 5, 6; Table 1). These genes present a robust and conserved expression across vertebrates. However, their roles and functions are still poorly understood and their expressions do not necessarily define domains *per se*. Therefore, caution has to be borne in mind during the following analysis.

**Table 1:** Microzone histogenetic codes of the shark basal hypothalamus corresponding to schemes in Figure 6.

	*ScNkx2.1*	*ScEmx2*	*ScOtp*	*Shh/ScShh*	*ScDlx2/5*	*ScLhx5*	*ScLmx1b*	*ScPitx2*	*ScPitx3a*	*ScFoxa1*	*ScFoxa2*	*ScNeurog2*
BAt1	+	−	+	−	−	+	−	−	−	−	−	−
BAt2	+	−	+	−	−	−	−	−	−	−	−	−
BAt3	+	−	−	−	+	−	−	−	−	−	−	−
BAt4	+	−	−	−	+*	−	−	−	−	−	−	−
BAt5	+	+	−	−	+*	−	−	−	−	−	−	−
BAt6	+	+	+	−	−	+	−	−	−	−	−	−
Tu1	+	−	−	−	−	+	−	−	−	−	−	−
Tu2	+	−	−	−	+	−	−	−	−	−	−	−
Tu3	+	−	−	+	−	+	−	−	−	−	−	−
Tu4	+	−	−	+	+	−	−	−	−	−	−	−
Tu5	+	−	−	−	+	−	−	−	−	−	−	−
Tu6	+	+	−	−	+	+	−	−	−	−	−	−
Rtu1	+	−	−	−	+	−	−	−	−	−	−	−
PM1	+	+	−	−	−	+	−	−	−	−	−	−
PM2	+	+	+	−	−	+	−	−	−	−	−	−
PRM1	+	+	−	−	−	+	−	−	−	−	−	−
PRM2	+	+	+	−	−	+	−	−	−	−	−	−
MM1	+	+	−	−	−	+	−	−	−	−	−	−
RM1	−	−	−	−	−	−	+	+	+	+	+	+
RM2	−	−	−	+	−	−	+	+	+	+	+	+
RM3	−	−	+*		−	+*	+	+	+	+	+	+

*Asterisks indicate that cells expressing this gene are not located in the ventricular zone. For abbreviations, see list*.

### Basal Acroterminal Domains

The acroterminal domain involves the alar and basal plate spreading from the rostral-most roof plate to the rostral-most floor plate. It has been suggested that specialized structures like the *lamina terminalis*, the optic chiasm and the neurohypophysis emerge here under particular signaling events (Puelles et al., 2012; Puelles and Rubenstein, 2015). We have identified at least 6 subdomains inside the basal acroterminal region named 1–6 from dorsal to ventral (BAt1–6; Figure 6B). Noteworthy the acroterminal territory (medial) is easily distinguishable from the remaining hypothalamus (lateral) due to genes differentially expressed at these locations (compare Figures 6A,B).

The two dorsal-most domains (BAt1–2) are positive for *ScNkx2.1* (Figure 1I) and *ScOtp* (Figure 2F) but only BAt1 shows expression of *ScLhx5* (Figure 3J). Furthermore, *ScOtp* co-distributes with *ScNkx2.8* (Figures 2F,F′). BAt1–2 subdomains are negative for other genes broadly expressed in the Tu such as *ScDlx2/5* and Shh (compare Figure 1M with Figure 2F). Of note, at later stages *ScLhx5* is absent from the midline (arrowhead in Figure 3K). Noteworthy, BAt1–2 are almost co-extensive with the developing adenohypophysis (Figures 1I, 6B), which in part is co-extensive with negative subdomains for *ScDlx2/5-*expression and Shh immunoreactivity (Figure 1M). Of note, these gaps are as wide as the adenohypophysis, which has been noted in other vertebrates even for different adenohypophysis sizes (see Figure 2N in Manning et al., 2006), suggesting a role for the adenohypophysis in the local patterning of the hypothalamus. In shark, both the gaps of *ScDlx2/5-*expression and Shh immunoreactivity and the expression of *ScNkx2.8* are wider than the medio-lateral extension of *ScOtp*-expression (Figures 2F,F′), which suggests the existence of additional medio-lateral subdomains.

BAt3 (the acroterminal region at the level of the neurohypophysis) is also Shh immunonegative and also expresses *ScNkx2.1* (Figures 1H,I), but differently from BAt1–2, it expresses *ScDlx2/5* (Figures 1L, 6B).

Ventrally to BAt3, we identified BAt4 as a subdomain that corresponds to the primourdium of the *saccus vasculosus* (Figure 6B; see also Van de Kamer and Shuurmans, 1953; Sueiro et al., 2007). The initial tiny domain expands becoming morphologically distinguishable (stage 29, Figures 1H, 2E; stage 30, Figure 1I). This domain is characterized by the expression of *ScNkx2.1* and dispersed *ScDlx2/5*-expressing cells (Figure 1L). Since *ScDlx2/5* is involved in the development of a GABAergic phenotype (Anderson et al., 1999), its expression in the *saccus vasculosus* could explain the existence of GABAergic cells at this point (Sueiro et al., 2007). Besides, GFAP-immunoreactivity has been described to be restricted to BAt4 (the developing *saccus vasculosus*), and it is not observable in more caudal subdomains (Sueiro et al., 2007). Finally, BAt4 is also characterized by lack of *ScEmx2* (Figure 3B) which, however, is present in more caudal acroterminal subdomains (Figures 3C,D; see also BAt5 in Figure 6B) and in lateral (non-acroterminal) domains (Figures 6A,D). Noteworthy, in *S. canicula*, the tip of the notochord has been described to reach the primordium of the *saccus vasculosus* (BAt4; Figure 1 in Van de Kamer and Shuurmans, 1953) suggesting a causal relationship to *saccus vasculosus* development.

BAt4 shares *ScNkx2.1* expression and dispersed *ScDlx2/5*-expressing cells with the domain ventral to it (BAt5; Figure 6B). However, as commented above, BAt5 differentially presents a lack GFAP-immunoreactivity and the presence of *ScEmx2* expression (Figures 3C,D; see also Figure 6B) at late stages of development.

**Figure 7:**
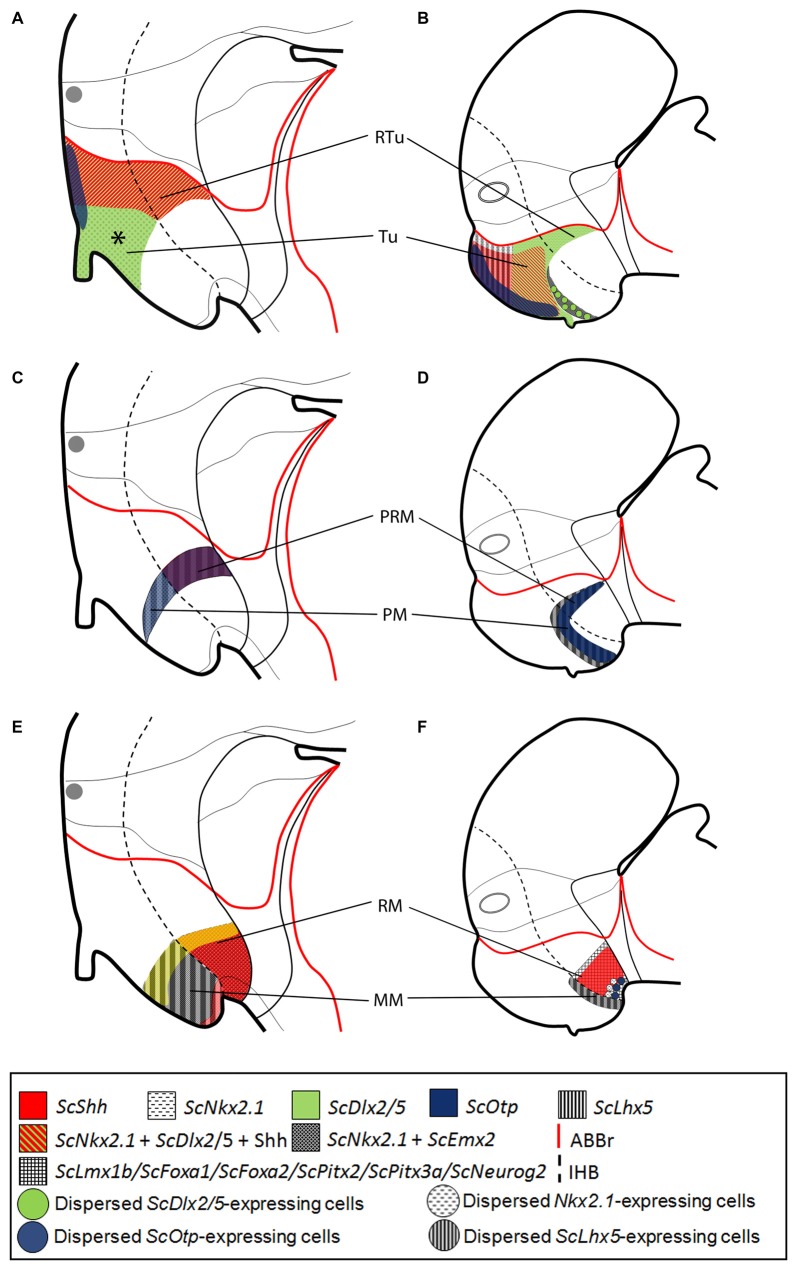
Comparative representations of microzones defined in mammals at E13.5 **(A,C,E)** and chondrichthyans at stage 29 **(B,D,F)** by sets of ortholog genes. Comparisons consider genes expressed in prosomeric histogenetic domains (**A,B**, Tu/RTu; **C,D**, PM/PRM; **E,F**, MM/RM). For simplicity schemes represent markers as in parasagittal sections but not in medial sagittal sections. **(A,B)** *Nkx2.1*, *Dlx2/5*, *Otp*, *ScShh/Shh* and *Lhx5* expression in the Tu/RTu. Though similar genes are expressed they define different microzones. In mammals *Lhx5* becomes dowregulated in the Tu/RTu. In the shark *Emx2* is also expressed in part of the Tu. **(C,D)** *Nkx2.1*, *Otp* and *Lhx5* are commonly expressed in the PM/PRM. Again, they define different subdomains. *Shh* is expressed in the PRM of mammals. *Emx2* is expressed in the whole PM/PRM. **(E,F)** *Nkx2.1*, *Emx2*, *Shh*, *Lmx1b*, *Foxa1*, *Foxa2*, *Pitx2*, *Pitx3* and *Neurog2* are expressed in MM/RM. They define different territories though a border between MM and RM seems to exist being more evident in chondrichthyans than in mammals. Data was obtained from the Allen Developing Mouse Brain Atlas (http://www.developingmouse.brain-map.com/) and the literature: *Shh*, *Nkx2.1*, *Dlx5*, *Otp* (Morales-Delgado et al., 2011, 2014; Puelles et al., 2012), *Lhx5* (Szabó et al., 2009; Abellán et al., 2010; Puelles et al., 2012), *Emx2* (Shimamura et al., 1995; Suda et al., 2001; Szabó et al., 2009), *Lmx1b* (Asbreuk et al., 2002; Martínez-Ferre and Martínez, 2012; Puelles et al., 2012), *Pitx2* (Martin et al., 2004; Puelles et al., 2012), *Foxa1* (Diez-Roux et al., 2011; Martínez-Ferre and Martínez, 2012; Puelles et al., 2012), *Foxa2* and *Neurog2* (Osório et al., 2010; Puelles et al., 2012). For abbreviations, see list.

The ventral-most acroterminal domain is BAt6 which express *ScNkx2.1*, *ScEmx2*, *ScOtp* and *ScLhx5*, but not *ScDlx2/5* (see Figure 6B). However, we cannot discard that this territory could be interpreted as the floor plate of the MM (Figure 6D) that in mouse (but not in shark; compare Figures 7E,F) differentially expresses *Shh* (see Figure 8.9B in Puelles et al., 2012).

### Tuberal (Tu/RTu) Subdomains

The dorsal and rostral-most domain is Tu1 and expresses genes like *ScNkx2.1* and *ScLhx5* (Figures 1H, 2A, 6C). Caudally to it, we have distinguished a similar domain lacking *ScLhx5* but expressing *ScDlx2/5*, named as Tu2 (compare 1L and Figure 2A; see Figure 6C). These two subdomains belong to the subliminal part of the basal hypothalamus and so, they lack *ScShh* (compare Figures 2I,J; see Figure 6C) and they express *ScNkx2.8* and *ScLhx9* (see Figure 5B in Santos-Durán et al., 2016). More ventrally, two subdomains, Tu3 and Tu4, appear as the ventral extension of Tu1 and Tu2 respectively, since they share with them either *ScLhx5* or *ScDlx2/5* expression. However, they additionally express *ScShh* (Figure 6C) but lack *ScNkx2.8* and *ScLhx9* expression (see Figure 5B in Santos-Durán et al., 2016). The expression of *ScLhx5* in the Tu1/Tu3 appears complementary to that of *ScDlx2/5* in Tu2/Tu4 (compare Figure 1L with Figure 2A; see Figure 6C). Complementary patterns between *Dlx* and *Lhx5* have been previously described in the mouse forebrain (Sheng et al., 1997), which suggests a conserved inhibitory relationship between both genes. Moreover, a small domain ventral (and caudal) to Tu4, which expressed *ScNkx2.1* and *ScDlx2/5* but was negative to Shh immunoreactivity, was referred as Tu5 (Figure 6C). A more ventral subdomain, referred as Tu6 is characterized by the expression of *ScEmx2* and a dispersed distribution of *ScDlx2/5*- and *ScLhx5*-expressing cells (compare Figure 1L with Figures 2D and Figure 3A; see also 6C).The dorsal and caudal-most subdomain identified is RTu1 (Figure 6C), which expresses the same genes as Tu2 and Tu5 (*ScNkx2.1*, *ScDlx2/5*; see Figure 6C).

### Perimamillar (PM/PRM) Subdomains

Ventral to Tu6, we identified PM1 a domain where *ScNkx2.1*/*ScEmx2*/*ScLhx5* are co-expressed (compare Figures 1K, 3C,K; see Figure 6D). We term PM2 (PM-like in Santos-Durán et al., 2015) the subdomain expressing *ScNkx2.1/ScEmx2/ScLhx5/ScOtp* (compare Figures 1K, 3C,K,L; see Figure 6D). The caudal continuation of PM1 and PM2 are referred as PRM1 and PRM2 (PRM-like in Santos-Durán et al., 2015) and express the same genes (Figure 6D).

### Mamillar (MM/RM) Subdomains

MM1 (MM-like in Santos-Durán et al., 2015) expresses *ScNkx2.1/ScEmx2/ScLhx5* (Figures 1K, 2D, 3A) but not *ScOtp* (Figures 2H, 6D). Of note, the genes expressed in the MM1 show a clear-cut border with those expressed in the RM domain (RM-like in Santos-Durán et al., 2015; see Figures 6D,E).

We have identified three dorso-ventral subdomains in the ventral and caudal-most point of the basal hypothalamus here referred as RM1, RM2 and RM3 (Figure 6E), which together fairly correspond to the previously defined RM-like territory (Santos-Durán et al., 2015; see also Figure 5). The dorsal-most domain, RM1, may be defined based on lack of Shh immunoreactivity at stage 29 (as does not reaches the ABB; see Santos-Durán et al., 2016) and the expression of *ScLmx1b/ScPitx2/ScPitx3a/ScFoxa1/ScFoxa2/ScNeurog2* (compare Figure 1A with Figures 3A–D; see Figure 6E). In the RM2, these genes co-distribute with Shh immunoreactivity while the ventral-most domain, RM3, is again characterized by the lack of Shh immunoreactivity (compare Figure 1F with Figures 1E–H; see Figure 6E). Of note, from stage 30 onwards, *ScPitx2* is expressed in the whole RM (Figures 4J,M) but the downregulation of other genes suggest the existence of even more dorso-ventral subdomains. Finally, RM3 also presents *ScLhx5*- and *ScOtp*-expressing cells likely born in neighbor domains (Figures 2D,H, 6E; Table 1).

### Evo-Devo Considerations Concerning the Basal Hypothalamus

**Figure 8:**
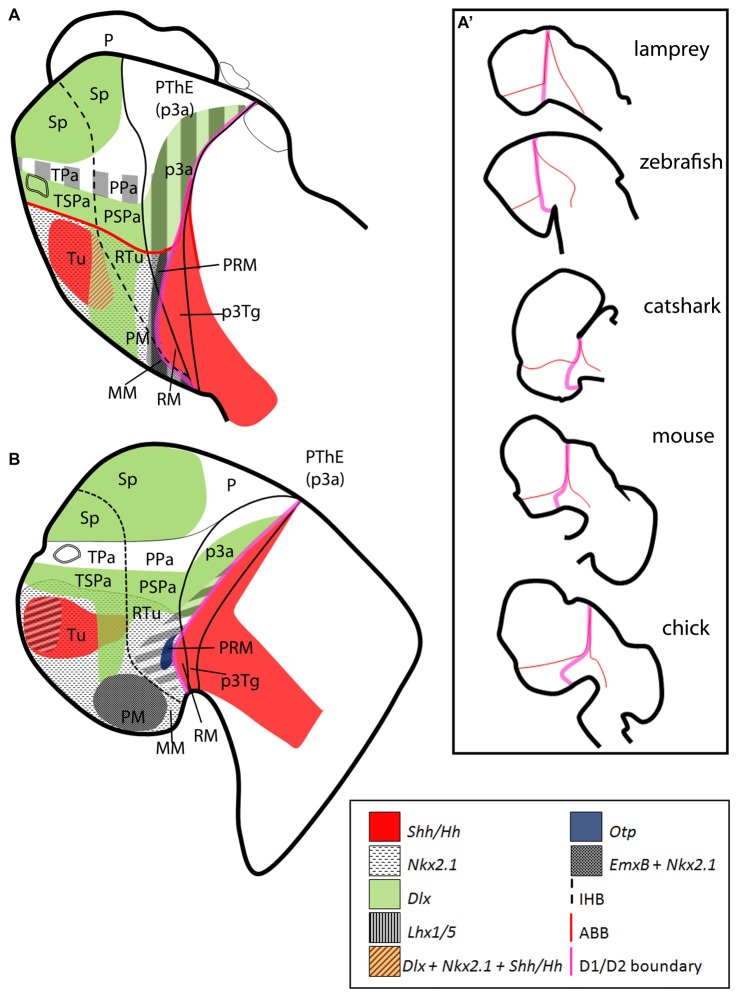
Sagittal schematic representations of the expression patterns of some orthologs here considered in **(A)** lamprey (adapted from Martínez-de-la-Torre et al., 2011) and **(B)** zebrafish (adapted from Hauptmann and Gerster, 2000). Schemes represent parasagittal sections at mid development. **(A′)** Conserved expression patterns suggest an alternative segmental border (pink line), which become deformed through evolution (adapted from Moreno and González, 2011). Data based on the following literature: **(A)** Murakami et al. (2001); Myojin et al. (2001); Ogasawara et al. (2001); Uchida et al. (2003); Osorio et al. (2005); Osório et al. (2006); Guérin et al. (2009); Tank et al. (2009); Kano et al. (2010); Martínez-de-la-Torre et al. (2011); Sugahara et al. (2011); **(B)** Barth and Wilson (1995); Hauptmann and Gerster (2000); Mathieu et al. (2002); Kapsimali et al. (2004); Jeong et al. (2006); Filippi et al. (2007, 2012); Ryu et al. (2007); Del Giacco et al. (2008); Osório et al. (2010); Yang et al. (2012); Lauter et al. (2013); Wolf and Ryu (2013); Manoli and Driever (2014). For abbreviations, see list.

The prosomeric model offers a key tool to study homologies and brain evolution (Puelles and Rubenstein, 2003, 2015). Counterparts of the genes here considered have been also studied in other vertebrates. Nevertheless, the lack of detailed data in prosomeric terms makes difficult to perform comparisons with most groups, except for mammals, at the level of microdomains. Below, detailed comparisons are made with mammals and we assume they are mostly transferable to other amniotes (Figure 7) due to the similarity of several patterns observed between mice and birds (Manning et al., 2006; Bardet et al., 2008, 2010; García-Calero et al., 2008; Abellán et al., 2010; also reviewed in Domínguez et al., 2014). Gross comparisons are also made with anamniotes that are, however, still informative (Figure 8).

#### Comparisons With Amniotes

In mouse, different works have addressed the expression of the orthologs here considered [*Shh*, *Nkx2.1*, *Dlx5*, *Otp* (Morales-Delgado et al., 2011, 2014; Puelles et al., 2012), *Lhx5* (Szabó et al., 2009; Abellán et al., 2010; Puelles et al., 2012), *Emx2* (Shimamura et al., 1995; Suda et al., 2001; Szabó et al., 2009), *Lmx1b* (Asbreuk et al., 2002; Martínez-Ferre and Martínez, 2012; Puelles et al., 2012), *Pitx2* (Martin et al., 2004; Puelles et al., 2012); *Foxa1* (Diez-Roux et al., 2011; Martínez-Ferre and Martínez, 2012; Puelles et al., 2012); *Foxa2*; and *Neurog2* (Osório et al., 2010; Puelles et al., 2012)] that are also available in the Developing Mouse Brain Atlas^1^. We compared these patterns between mouse stage 13.5 and shark stage 29.

In both, mouse and shark, the Tu/RTu is characterized by the expression of *Nkx2.1*, *Dlx2/5* and *Otp* (Figures 7A,B; see also Santos-Durán et al., 2015). However, differences in sub-compartmentation emerge while considering additional genes. In mouse, *Lhx5* is broadly expressed in the basal hypothalamus before stage 13.5 (Szabó et al., 2009; Abellán et al., 2010; Developing Mouse Brain Atlas). Nevertheless, after this stage, it becomes downregulated and restrictedly expressed in non-Tu/RTu domains (Figure 7A). In shark, *ScLhx5* is expressed in the dorsal-most Tu from stage 29 to 31 (Figure 7B). Besides, in the mouse, the expression of *Shh* is restricted to the dorsal and caudal-most Tu and RTu (Figure 7A) while in *S. canicula* it is fairly expressed in two subdomains of Tu but absent in the RTu (Figure 7B). Noteworthy, the differential spatial distribution of Shh between both models leads to the emergence of a subdomain in the mouse Tu (asterisk in Figure 7A) containing *Dlx2/5* alone, apparently not present in shark (Figure 7B). However, a careful view suggests that this domain could correspond to the reduced Tu5 domain found in shark (see Figure 6C), so that in mouse this *Dlx2/5*-expressing domain could have been expanded at expenses of Shh-expressing domains due to changes in ventro-caudal signaling. Finally, in mouse, *Emx2* is absent from the Tu/RTu while in shark it is expressed in Tu6 (Figures 6C, 7A,B).

The PM/PRM is characterized in both models by *Otp* and *Lhx5* expression (Figures 7C,D). Of note, in shark, this compartment can be subdivided in a rostral PM1/PRM1 that lacks *ScOtp* and a caudal PM2/PRM2 that does express this gene. In shark, both compartments also express Sc*Emx2* (Figures 7C,D). Noteworthy, the PRM of shark lacks *Shh* expression in contrast to mouse (Figures 7C,D).

In the MM/RM, in both species genes expressed in the RM, like *Lmx1b* or *Foxa1*, appear to form a clear-cut border of expression with respect to those expressed in the MM (and even in the PRM) as *Nkx2.1*, *Emx2* and *Lhx5* (see Figures 7E,F). This seems to be a conserved feature across vertebrates (see below). However, in mouse, genes like *Pitx2* and *Neurog2* are expressed in both domains (Figures 7E,F) though *Neurog2* is restricted to RM at earlier stages (termed as p3Tg in Figures 2B,B′ in Osório et al., 2010).

Together, this analysis reveals at least two things. First, one to one comparisons are useful to understand how interspecific variability emerges. Interestingly, the same set of homologous genes defines new or different microdomains among different species. Second, the number of microdomains identified increase with the number of genes analyzed, though their significance in terms of homology (common ancestry) becomes elusive. This raises non-trivial questions concerning homology establishment (Abouheif, 1997; Puelles and Medina, 2002) and compel us to consider other possible interpretations in the context of the prosomeric model.

#### Comparisons With Anamniotes

Many of the genes here considered have been already studied in agnathans and teleosts (Figure 8). Though it is difficult to establish one to one comparisons at the level of subdomains, common traits do exist between these groups (including chondrichthyans). Therefore, such characters are assumed to be transferable to other anamniotes.

In amniotes, genes expressed in the *Nkx2.1*-expressing hypothalamus (*Emx2*, *Otp*, *Lhx5*) abut those expressed more ventro-caudally (*Shh*, *ScPitx2*, *ScPitx3*, *ScNeurog2* and *ScLmx1b*). Furthermore, the last group of genes describes a continuous line from the floor plate of the terminal hypothalamus and extends into the zli (Figures 6A,C and Figure 8). Such abutted expression is typical of segmental boundaries (for definition of segmental boundaries see Dahmann et al., 2011; Cavodeassi and Houart, 2012; Kiecker and Lumsden, 2012; see also Larsen et al., 2001; Puelles et al., 2012) and has not been observed at other points of the caudal secondary prosencephalon. Having this in mind we decided to look for other evidences for segmental boundaries at this point as reduced cell proliferation and the presence of signaling centers. Noteworthy, at least in sharks, reduced cell proliferation (PCNA-negative cells at the ventricular zone in Figures 3E–H) can be also detected bordering the domain where Shh and other markers are expressed in the caudal hypothalamus. Moreover, transverse *Wnt* signals seem to describe such border from the zli to the MM/RM boundary in different vertebrates (Figure 8A′; see also Guérin et al., 2009; Quinlan et al., 2009) while the situation in mice remain unclear. Finally, such border also seems to be the same as that delineated by Figdor and Stern (1993) between segment D1 and D2. Noteworthy, in the lamprey (Figure 8A), the expression of *Wnt* signals resemble that of other *Wnt* genes between rhombomers (Riley et al., 2004) an idea already suggested in Santos-Durán (2015). Though our results suggest that such border could get deformed on the course of evolution as shown in Figure 8A′, these evidences are not necessarily supported in mice where the expression of genes like *Neurog2* and *Pitx2* is continuous through the MM/RM rather than restricted to the RM (Figure 7). Of note, a recent review on vertebrate forebrain development also suggests the existence of a novel secondary organizer at this point (Puelles, 2017) as previously suggested in Santos-Durán (2015).

However, the idea that the currently identified as MM/RM border could indeed represent the hypothalamic-diencephalic border implies a new model of the hypothalamus that would require re-examination of other postulated limits and thus deserves further investigation.

## Conclusion

This work belongs to a series of articles (Santos-Durán et al., 2015, 2016) addressing the development and evolution of the chondrichthyan hypothalamus and also the new proposals of the prosomeric model (Puelles et al., 2012; Puelles and Rubenstein, 2015) on the mentioned region.

Here, our combinatorial analysis revealed the existence of many different microdomains within the main subdomains of the prosomeric basal hypothalamus of the shark (Tu/RTu; PM/PRM; MM/RM). The genes considered in this study (*ScOtp*, *ScDlx2/5*, *ScNkx2*.1, *ScShh*, *ScLhx5, ScEmx2, ScLmx1b, ScPitx2, ScPitx3a, ScFoxa1, ScFoxa2 and ScNeurog2*) are well conserved in vertebrates. However, detailed comparisons at the level of microdomains under the prosomeric framework can be only performed with mammals, on which abundant data are available. Such analysis reveals a number of microzones that do not exactly fit those described in mice (Ferrán et al., 2015). Understanding the homology and evolution of such microdomains results daunting and can be misleading. However, these results illustrate, at least in part, how organisms became different in spite of expressing similar set of homologous genes.

## Author Contributions

GNS-D, IR-M and EC designed the study and analyzed the data. SM contributed to data acquisition. GNS-D, AM and SF-G performed the experiments. GNS-D wrote the manuscript with inputs from all authors.

## Conflict of Interest Statement

The authors declare that the research was conducted in the absence of any commercial or financial relationships that could be construed as a potential conflict of interest. The reviewer NM and handling Editor declared their shared affiliation, and the handling Editor states that the process nevertheless met the standards of a fair and objective review.

## Footnotes

^1^http://developingmouse.brain-map.org/

## Abbreviations

ABB: alar-basal boundary
Ah: adenohypophysis
AHy: alar hypothalamus
ap3: prosomere 3, alar part
BAt: basal acroterminal subdomain
HDB: hypothalamic-diencephalic border
hp1: prosomere hp1 or peduncular
hp2: prosomere hp2 or terminal
IHB: intrahypothalamic border
MM: mamillary area
Nh: neurohypophysis
P: pallium
p3Tg: prosomere 3, tegmental part
Pa: paraventricular area
PM: perimamillary area
PPa: paraventricular area, peduncular part
PRM: periretromamillary area
PSPa: subparaventricular area, peduncular part
PThE: prethalamic eminence (ap3)
RM: retromamillary area
RTu: retrotuberal area
Sp: subpallium
SPa: subparaventricular area
Sv: saccus vasculosus
T: telencephalon
TPa: paraventricular area, terminal part
TSPa: subparaventricular area, terminal part
Tu: tuberal area
zli: zona limitans intrathalamica.
